# Oxidative agents elicit endoplasmic reticulum morphological changes suggestive of alterations in lipid metabolism

**DOI:** 10.17912/micropub.biology.000462

**Published:** 2021-09-20

**Authors:** Alba Torán-Vilarrubias, María Moriel-Carretero

**Affiliations:** 1 Institut de Génétique Humaine (IGH), Université de Montpellier, Centre National de la Recherche Scientifique, 34396 Montpellier CEDEX 05, France; 2 Centre de Recherche en Biologie cellulaire de Montpellier (CRBM), Université de Montpellier, Centre National de la Recherche Scientifique, 34293 Montpellier CEDEX 05, France

## Abstract

The endoplasmic reticulum (ER) is a central organelle in charge of correct protein folding; lipids synthesis, modification, and sorting; as well as of maintenance of calcium homeostasis. To accomplish these functions, the ER lumen possesses an oxidative potential. Challenging cells with reductive agents therefore provokes an ER stress that immediately affects protein folding, and which morphologically manifests by an expansion of the cytoplasmic ER network. Yet less is known about the impact on the ER of exposing cells to oxidative agents, which risk to exacerbate the basal, physiologically oxidative environment. We have monitored the morphology of the ER of *Saccharomyces cerevisiae* in response to this type of treatment. We bring the notion that oxidative agents give rise to diverse alterations in the perinuclear ER subdomain that are suggestive of lipid metabolism perturbations.

**Figure 1. Characterization of the effect of oxidative agents on the endoplasmic reticulum morphology:**
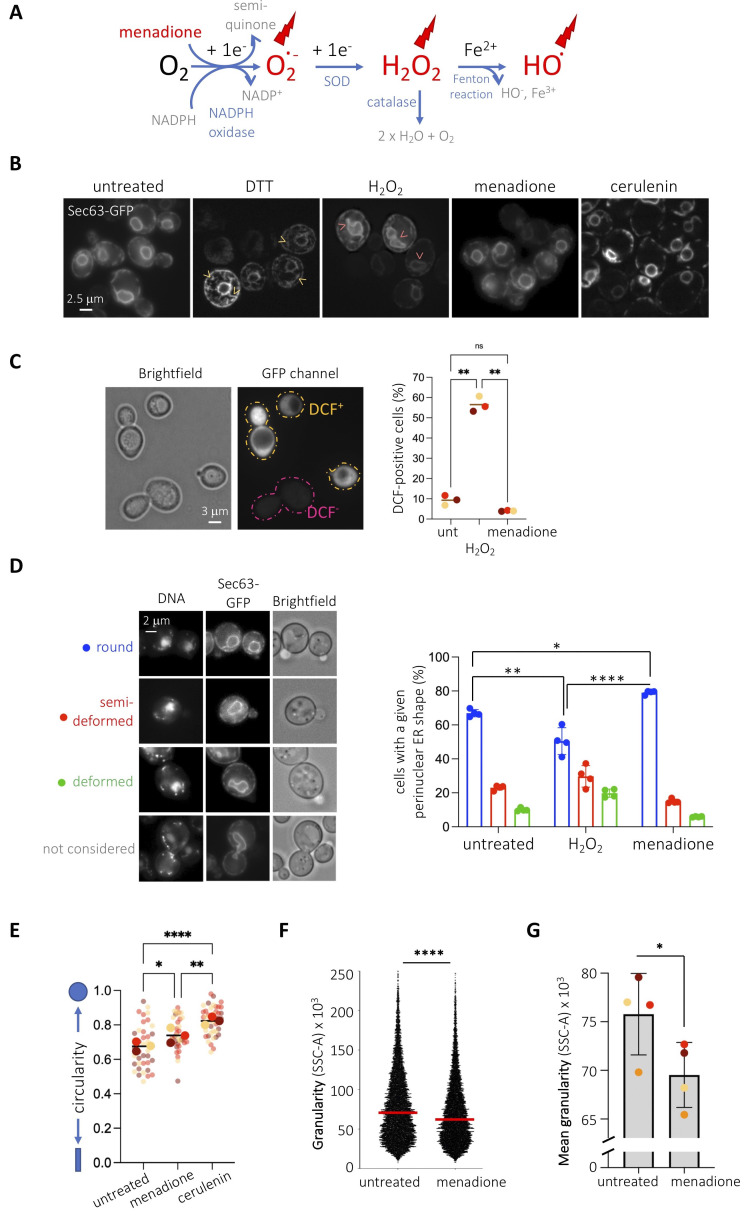
**(A)** Reactive Oxygen Species (ROS, in red) include **superoxide anion (O^.-^_2_)**, generated from NADPH oxidation, which gives rise to **hydrogen peroxide (H_2_O_2_)** after superoxide dismutase (SOD) acts as catalyst, which can then give rise to the highly reactive **hydroxyl ion (HO^.^)** through a Fenton reaction. Menadione is mentioned in red as it can act as a one-electron donor, which yields a semiquinone and one **superoxide anion (O^.-^_2_)**. After ROS exhaust the cell scavenging system, which includes the action of catalase, they become available to harm the cellular machinery (red bolts). **(B)** WT cells transformed with a plasmid expressing Sec63p-GFP were untreated or treated for 2h with 8 mM DTT, 10 mM H_2_O_2_, 1 mM menadione or 10 µg/mL cerulenin. Yellow arrows point at overgrown cytoplasmic ER membranes. Pink arrows point at deformed perinuclear ER membranes. **(C)** The molecular probe 2’,7’-dichlorodihydrofluorescein diacetate (H_2_DCFDA) was used to measure hydrogen peroxide levels in live, full cells in order to check that the used oxidizing agents were leading to the expected effects. This probe diffuses into cells and is naturally deacetylated to yield H_2_DCF. H_2_O_2 _molecules oxidize it to dichlorofluorescein (DCF), which emits fluorescence at 522 nm (McLennan and Esposti 2000). Treating the cells with hydrogen peroxide leads to a neat increase in the percentage of fluorescent cells in the population. In the case of menadione treatment, the increase in superoxide anions (Figure 1A) is reported to trigger an upregulation of catalase activity, which translates into a detoxification (thus decrease) in hydrogen peroxide molecules (Jamieson 1992; Oh *et al.* 2000). We detect this as a mild but consistent decrease in the percentage of fluorescent cells. The graph shows the percentage of cells displaying cytoplasmic fluorescence. Mean values +/- standard error of the mean out of three independent experiments are plotted. At least 150 cells were counted per condition and experiment. The asterisks reveal the significant difference of the means after applying a one-way ANOVA. **, *p*<0.01; ns, non-significant. **(D, left panel)** The perinuclear ER patterns observed in (B) were arbitrarily classified as shown. Prior to imaging, cells were incubated for 20 min with 4 µg/mL DAPI to visualize the nuclear DNA. “Brightfield” shows the whole cell. **(D, right panel)** Quantitative analysis of perinuclear (pn)ER morphology of cells presented in (B) using the indicated criteria. Percentages of cells displaying each pnER phenotype are shown. Mean values +/- standard error of the mean out of four independent experiments are plotted. At least 150 cells were counted per condition and experiment. The asterisks reveal the significant difference of the means after applying a one-way ANOVA. *, *p*<0.05; **, *p*<0.01; ****, *p*<0.0001. **(E)** The perimeters of nuclei as defined by Sec63p-GFP signals from images as those presented in (B) from three independent experiments were defined with ImageJ and their circularity assessed. To illustrate reproducibility, the graph shows a Super-Plot (Lord *et al.* 2020) in which semi-transparent dots from each experiment are shown in a different color and the mean from each of them is superimposed in full color. The mean of the means is indicated by a black bar. The asterisks reveal the significant difference of the means after applying a one-way ANOVA. *, *p*<0.05; **, *p*<0.01; ****, *p*<0.0001. **(F)** Cells as described in (B) were analyzed by flow cytometry. Side-Scattered light (SCC-A, granularity) of the cells is presented for one single experiment, where n > 10.000 events and median values are indicated by a red line. The asterisks inform on the significant difference between the populations after performing a nonparametric Mann-Whitney test, for which *p*<0.0001. **(G)** The mean value from each independent experiment as the one presented in (F) was plotted in a different color to illustrate reproducibility. The asterisk and the *p*-value inform on the significant difference between the means after performing a paired *t*-test, where *p*=0.0137. No solvent is added to the “untreated” condition since menadione and hydrogen peroxide were prepared in water.

## Description

Our laboratory studies the relationship between genotoxic stress and the endoplasmic reticulum (ER). Since multiple genotoxins are reported to generate oxidative stress (Mizumoto *et al.* 1993; Singh and Xu 2016), we wanted to establish the impact of *bona-fide* oxidative agents on basal ER morphology as a control before further studies. To this end, we chose two agents, menadione and hydrogen peroxide, whose action mode relies on different reactive oxygen species (Figure 1A and (Castro *et al.* 2008; Roscoe and Sevier 2020)). We visualized the ER of *Saccharomyces cerevisiae* cells by transforming them with a plasmid expressing the transmembrane ER protein Sec63p tagged with GFP at its C-terminus (Prinz *et al.* 2000). Under basal conditions, this tool permits to see a central ring, the perinuclear ER (also known as the outer nuclear membrane), the cortical ER under the plasma membrane and eventual connections between both, the cytoplasmic ER (Figure 1B). We treated cells with the reducing agent dithiothreitol (DTT) to trigger a *bona-fide* unfolded protein ER stress which, as expected, gave rise to an overgrowth of cytoplasmic ER membranes, a response aimed at increasing the challenged ER protein folding capacity (Ron and Walter 2007). When cells were exposed to 10 mM hydrogen peroxide, whose entry in the cells was validated by an increase in fluorescence when using the probe 2’,7’-dichlorodihydrofluorescein diacetate (H_2_DCFDA) (Figure 1C), we did not find any alteration in the cytoplasmic ER, but a deformation in the perinuclear ER subdomain (Figure 1B). Alterations in shape at the perinuclear ER are suggestive of lipid rather than protein ER stress (Santos-Rosa *et al.* 2005; Witkin *et al.* 2012). To quantify this phenomenon, we established three degrees of deformation, which transitioned from almost perfectly spherical, to semi-deformed, to deformed (Figure 1D). This classification intentionally excludes cells in G2 / M, for which the nuclear membrane appears distorted because of cell division (Figure 1D). In comparison with the untreated condition, in which a 65% of cells display round nuclear shapes, hydrogen peroxide made this value significantly decrease down to 50% (Figure 1B & 1D, right). Next, we exposed cells to menadione, whose oxidative effect, mainly exerted by superoxide anions (Figure 1A), was also indirectly validated using the probe 2’,7’-dichlorodihydrofluorescein diacetate (H_2_DCFDA) (Figure 1C). Menadione also triggered a modification exclusively at the perinuclear ER but, in striking contrast, it was integrally in the opposite direction than hydrogen peroxide: the perinuclear ER displayed a perfectly spherical shape in 80% of the population (Figure 1B & 1D, right). This phenomenon mimics the effect of cerulenin (Figure 1B), an inhibitor of fatty acid synthase (Inokoshi *et al.* 1994) in whose presence phospholipid synthesis decreases thus nuclear membranes are incapable of expansion (Schneiter *et al.* 1996; Yam *et al.* 2011). In agreement, we confirmed that cells treated with menadione or cerulenin possess nuclei with increased circularity (Figure 1E). For the same reason, cerulenin induces the consumption of lipid stores, thus decreasing the number of lipid droplets in *S. cerevisiae* (Jacquier *et al.* 2011). To reinforce the notion that menadione limits lipid biosynthesis, we measured the granularity of cells as a readout for their content in lipid droplets and membranes (Suzuki *et al.* 1991; Lee *et al.* 2004). In agreement, we found that menadione decreases cell granularity, as established by flow cytometry (Figure 1F), and this in a very reproducible manner (Figure 1G).

In sum, we report that two oxidative stressors elicit morphological changes in the ER reminiscent to lipid rather than unfolded protein stress. Yet, hydrogen peroxide reactive oxygen species trigger nuclear membrane expansion or deformation while superoxide anion species match an extremely circular morphology, suggestive of an anti-lipogenic phenotype. This is in striking agreement with a recent report in which menadione was described to exert an anti-adipogenic activity by repressing the expression of multiple enzymes involved in the lipogenic program, among which fatty acid synthase (Funk *et al.* 2021). Our data therefore set the bases to further explore the molecular link between oxidative and lipid ER stresses.

## Methods

*Saccharomyces cerevisiae* cells were grown at 25°C in selective YNB liquid medium supplemented with 2% glucose in the absence of uracil to ensure plasmid maintenance. All experiments were performed with exponentially growing cells. To acquire side-scattered light values by flow cytometry, 430 μL of culture samples at 10^7^ cells/mL were fixed with 1 mL of 100% ethanol. Cells were centrifuged for 1 minute at 16000g and resuspended in 500 μL 50 mM Na-Citrate buffer containing 5 μL of RNase A (10 mg/mL, Euromedex, RB0474) for 2 hours at 50°C. 6 μL of 20 mg/mL Proteinase K (Euromedex, EU0090-C) were added for 1 hour at 50°C. Aggregates of cells were dissociated by brief sonication. 20 μL of this cell suspension were incubated with 200 μL of 50 mM Na-Citrate buffer containing 4 μg/mL Propidium Iodide (FisherScientific). Data were acquired on a MACSQuant Analyser X (Miltenyi Biotec) and analyzed with FlowJo software. For microscopy analyses, 1 mL of the culture of interest was centrifuged; then, the supernatant was thrown away and the pellet was resuspended in the remaining 50 μL. Next, 3 μL of this cell suspension was directly mounted on a coverslip for immediate imaging of Sec63p GFP-tagged protein signals. To measure cellular levels of hydrogen peroxide, cells were incubated with 10 µM H_2_DCFDA for 1 h prior to harvesting and washed twice with 1x PBS prior to imaging. Fluorescent signals were detected using the adequate wavelength and acquired with a Zeiss CCD AxioCam MRm monochrome camera from a Zeiss AxioImager Z1 microscope with ApoTome technology using a 40× Plan Apochromat 1.3-NA oil objective lens and Zen software. Images were acquired at 20–23 °C. Subsequent image visualization and analysis were performed with Image J v2.0.0-rc-69/1.52i. The determination of the percentage of different ER configurations was done through visual inspection by the experimenters. To quantify circularity of nuclei, Sec63p-GFP images were thresholded using the algorithm Otsu in ImageJ, and circularity measured using the command “shape descriptors”. Visual inspection by the experimenter of selected particles compared to the original images ensured that only real nuclei were considered. GraphPad Prism was used to plot and statistically analyze the results.

## Reagents

The wild-type strain (MM-35) is a W303 strain corrected for the *RAD5* gene. We used cerulenin (SC-200827A, Santa Cruz Biotechnology), DAPI (D9542, Sigma-Aldrich), DTT (10197777001, Sigma-Aldrich), menadione (M5750-25G, Sigma-Aldrich), H_2_O_2 _(H1009, Sigma-Aldrich) and H_2_DCFDA (35845, Sigma-Aldrich). pJK59 (*CEN-URA3-SEC63promoter-SEC63*-GFP(S65T,V163A)), used to visualize the ER, was a kind gift from Dr. Sebastian Schuck.
